# Comparison of Intra-Operative Vital Sign Changes during Total Thyroidectomy in Patients with Controlled and Uncontrolled Graves’ Disease

**DOI:** 10.3390/jcm7120566

**Published:** 2018-12-19

**Authors:** Hyeong Won Yu, In Eui Bae, Su-jin Kim, Young Jun Chai, Jae Hoon Moon, Jung-Hee Ryu, Ah-Young Oh, June Young Choi, Kyu Eun Lee

**Affiliations:** 1 Department of Surgery, Seoul National University Bundang Hospital, 82, Gumi-ro 173 Beon-gil, Bundang-gu, Seongnam-si, Gyeonggi-do 13620, Korea; hyeongwonyu@gmail.com (H.W.Y.); inniggo@gmail.com (I.E.B.); 2 Department of Surgery, Seoul National University Hospital and College of Medicine, 101 Daehak-ro, Jongno-gu, Seoul 03080, Korea; su.jin.kim.md@snu.ac.kr (S.-j.K.); kyueunlee@snu.ac.kr (K.E.L.); 3 Department of Surgery, Seoul National University Boramae Medical Center, 20 Boramae-ro 5-gil, Dongjak-gu, Seoul 07061, Korea; kevinjoon@naver.com; 4 Department of Internal Medicine, Seoul National University Bundang Hospital, 82, Gumi-ro 173 Beon-gil, Bundang-gu, Seongnam-si, Gyeonggi-do 13620, Korea; jaemoon76@gmail.com; 5 Department of Anesthesiology & Pain Medicine, Seoul National University Hospital and College of Medicine and Seoul National University Bundang Hospital, 82, Gumi-ro 173 Beon-gil, Bundang-gu, Seongnam-si, Gyeonggi-do 13620, Korea; jinaryu74@gmail.com (J.-H.R.); oay1@snubh.org (A.-Y.O.)

**Keywords:** thyroid storm, vital sign change, Graves’ disease, uncontrolled Graves’ disease, thyroidectomy

## Abstract

Thyroid storm (TS) is a life-threatening emergency endocrine condition. Thyroid hormones should be normalized before thyroidectomy is performed in patients with Graves’ disease. However, thyroid hormone levels are inevitably high in patients undergoing surgery. This study analyzed differences in vital sign changes during thyroidectomy between patients with controlled and uncontrolled Graves’ disease and assessed thyroid hormone cutoffs for TS. Preoperative levels of the thyroid hormones free T4 (FT4), T3, and thyroid stimulating hormone (TSH) were retrospectively analyzed in patients who underwent total thyroidectomy for Graves’ disease. Patients were divided into those with uncontrolled Graves’ (UG) disease, defined as preoperative TSH <0.3 µIU/mL and FT4 >1.7 ng/dL, those with controlled Graves’ (CG) disease, those with extremely uncontrolled Graves’ (EUG) disease, defined as TSH <0.3 µIU/mL and FT4 >3.4 ng/dL, and finally, those without EUG (non-EUG). The 29 patients with Graves’ disease included 12 with CG group and 17 with UG. FT4 and T3 concentrations were significantly higher in the UG group. There were no differences in vital sign and anesthetic agent. These 29 patients could also be divided into those with (*n* = 4) and without EUG (*n* = 25). The mean age was lower (21.5 vs. 40.9 years, *p* < 0.001) and the mean operation time was shorter (121.4 vs. 208.8 min, *p* = 0.003) in the EUG group. Requirements for anesthetic agents were greater in the EUG group. Mean FT4 concentration in the EUG group was 3.8 ng/dL, and there were no changes in vital signs during surgery. Vital sign change during thyroid surgery was not observed in patients with uncontrolled Graves’ disease up to the twice upper normal limit of T4 level.

## 1. Introduction

Graves’ disease, caused by the overexpression of thyroid stimulating immunoglobulin, results in thyroid enlargement and increased levels of thyroid hormones [1,2]. Clinical characteristics of Graves’ disease include hyperthyroidism, a goiter with a neck mass, and ophthalmopathy [3]. The primary treatment of Graves’ disease is with anti-thyroid drugs (ATDs) or radioactive iodine (RAI) to maintain normal levels of thyroid hormones [4]. Another alternative is thyroidectomy. Guidelines recommend that patients undergo thyroidectomy only after attaining normal ranges of thyroid hormone levels by taking ATDs. However, surgery may be performed on some patients with high thyroid hormone levels [5]. These patients may experience adverse reactions to ATDs, including agranulocytosis, hepatotoxicity or urticarial; they may be unresponsive to ATDs, or may be pregnant women who refuse high-dose ATDs, or patients who experience goiter discomfort and want a quick operation, precluding normalization of thyroid hormone levels prior to surgery [6,7]. 

High thyroid hormone levels may cause thyroid storm (TS) [8], a life-threatening emergency condition first described in 1926 and characterized by multi-organ failure resulting from excessive secretion of thyroid hormones [9,10]. TS occurs frequently after infection, surgery, trauma, radioiodine treatment, or discontinuation of ATDs in patients with hyperthyroidism who are not properly treated [11,12]. TS initially presents with sudden high fever, tachycardia, hypertension, dyspnea, cardiac fibrillation, and arrhythmia [13]. Patients with TS may also experience gastrointestinal symptoms, including nausea, vomiting, diarrhea, abdominal pain, hepatomegaly, and jaundice, along with neurological symptoms such as emotional disturbance, electrolyte abnormalities, and decreased consciousness [13]. Studies in Japan have reported mortality rates as high as 11% [14]. However, most studies of TS have consisted of case reports, with no large case series reported to date. Because the incidence of TS is only 0.2 per 100,000 people [10], few physicians have encountered this condition. Thus, cutoff values for thyroid hormones and the incidence of TS during surgery have not been determined [5]. It is thus necessary to investigate changes in vital signs that occur during thyroidectomy in Graves’ disease patients with high thyroid hormone levels, and to determine thyroid hormone cutoff levels associated with TS. 

## 2. Materials and Methods

### 2.1. Patients

The medical records of patients with Graves’ disease who underwent total thyroidectomy from January 2014 to April 2017 at Seoul National University Bundang Hospital, Seoul, Korea, were retrospectively reviewed. This study was approved by the Research Ethics Committee (B-1801/445-113). Data analyzed included patient age, sex, body mass index (BMI), types and doses of ATDs, uses of β-blockers and lugol solution, and preoperative levels of thyroid hormones, including free thyroxine (FT4), triiodothyronine (T3), thyroid stimulating hormone (TSH), and thyroid stimulating hormone receptor antibodies (TSH-R-Ab). Other factors analyzed included complete blood count (CBC), liver enzyme concentrations (alanine transaminase, aspartate transaminase), type and duration of surgery, and types and doses of anesthetic agents (propopolol, remifentanil, nicardipine, and esmolol). Vital signs analyzed included systolic blood pressure (SBP), diastolic blood pressure (DBP), heart rate (HR), and body temperature (BT). Other factors included the occurrence of TS and complications such as agranulocytosis, hepatotoxicity, rash, urticaria, joint pain, and fever. 

### 2.2. Patient Management

FT4, T3, TSH, liver enzymes, and CBC were measured in patients diagnosed with Graves’ disease 4–6 weeks after taking propylthiouracil (PTU), methimazole (MMI), and carbimazol [7]. Patients with symptoms of palpitation were also treated with β-blockers. Treatment with ATDs was aimed at maintenance for 18 months. However, ATDs were discontinued and thyroidectomies performed when complications such as agranulocytosis, hepatotoxicity, rash, urticaria, arthralgia, and fever were observed or when the dosage of ATDs had to be continuously increased. Women who wanted to become pregnant, patients with airway obstruction symptoms, and patients with thyroid cancer who required an immediate operation were also treated surgically. The choice of open or robotic surgery was determined by the surgeon after consultation with the patient.

### 2.3. Definitions of Controlled and Uncontrolled Graves’ Disease

Reference ranges of thyroid hormones are 0.89–1.79 ng/dL for FT4, 81–197 ng/dL for T3, 0.3–4.0 µIU/mL for TSH, and 0.3–1.22 IU/L for TSH-R-Ab. Uncontrolled Graves’ (UG) disease was defined as preoperative FT4 >1.7 ng/dL and TSH <0.3 µIU/mL, with all other patients defined as having controlled Graves’ (CG) disease. In addition, patients were divided into those with extremely uncontrolled Graves’ (EUG) disease, defined as those with FT4 >3.4 ng/dL and TSH <0.3 µIU/mL, and those without EUG (non-EUG). 

### 2.4. Anesthetic Management

Standard monitoring was done with electrocardiography, noninvasive blood pressure measurement, and pulse oximetry after premedication with intravenous midazolam (0.03 mg/kg). Total intravenous anesthesia with propofol and remifentanil was used for induction and maintenance of anesthesia using target-controlled infusions (Orchestra infusion pump system (Fresenius vial, Brezins, France)). Rocuronium in doses of 0.6 mg/kg was used to facilitate muscle relaxation for endotracheal intubation, and additional doses of rocuronium 0.15 mg/kg were administered during surgery. After surgery and extubation, the patients were transferred to the post-anesthesia care unit.

During the operation, blood pressure was measured every 2 min and adjusted within ±20% that of medical ward administering small amounts of esmolol (5–10 mg), nicardipine (0.5–1 mg), labetalol (5 mg), ephedrine (5 mg) or phenylephrine (30 μg). If hypertension occurred, an intravenous bolus of labetalol or nicardipine was given. If hypotension occurred, a small amount of ephedrine or phenylephrine was administered. Tachycardia (HR >100 beats/min) was treated with esmolol or labetalol. 

### 2.5. Statistical Analysis

Continuous variables were expressed as means ± standard deviations and compared using Student’s *t*-tests. Categorical variables were expressed as numbers and percentages and compared by Pearson’s Chi-squared tests. Differences were considered significant at *p* values <0.05. All statistical analyses were performed using Statistical Package for the Social Sciences (SPSS, version 22.0.0, IBM, Armonk, NY, USA) for Windows. 

## 3. Results

### 3.1. Demographic and Clinical Characteristics of Patients

Of the 29 patients included in the study, 12 were assigned to the CG group and 17 to the UG group. Table 1 shows the demographic and clinical characteristics of these two groups. Mean age was similar in the CG (39.4 years) and UG (37.4 years) groups, and there was no statistically significant difference in sex and BMI. Two patients in the CG group and four in the UG group underwent robotic surgery. Mean operation times were similar in the CG and UG groups (123.8 vs. 140.3 min, *p* = 0.450). Serum concentrations of T3 (159.3 vs. 247.5 ng/dL, *p* = 0.009) and FT4 (1.2 vs. 2.6 ng/dL, *p* < 0.001) differed significantly in the CG and UG groups, whereas TSH (0.6 vs. 0.1 µIU/mL) and TSH-R-Ab concentrations did not. Ten patients in each group were treated with MMI, one in each group with carbimazole and one in each group with PTU. Eight patients in the UG group (47.1%), but none in the CG group, were administered β-blockers before surgery (*p* = 0.005); however, the use of lugol solution did not differ in these groups.

### 3.2. Intra-Operative Changes in Vital Signs and Use of Anesthetic Agents

Table 2 shows changes in vital signs and use of anesthetic agents by the two groups. There were no significant differences between the CG and UG groups in the incidence of SBP increase to >140 mmHg, the highest average SBP, the highest average DBP, and the highest SBP (151 mmHg vs. 159 mmHg) during surgery. The incidence of HR increases to >100 bpm, the highest average HR, and the highest HR (110 bpm vs. 127 bmp) were also similar in the two groups. None of the patients experienced a body temperature >38 °C, with the highest body temperatures in the CG and UG groups being 36.6 °C and 36.8 °C, respectively. There were no between-group differences in the total amounts of propofol and remifentanil during surgery, and no differences in the amounts of esmolel, nicardipine, labesin, and phenylephrine administered during surgery for transient blood pressure control. All patients had stable vital signs after surgery and were within the normal range.

### 3.3. Patients with Extremely Uncontrolled and Non-Extremely Uncontrolled Graves’ Disease

Table 3 shows the results in the EUG (*n* = 4) and non-EUG (*n* = 25) groups. Patients in the EUG group were significantly younger than those in the non-EUG group (21.5 years vs. 40.9 years, *p* < 0.001), but there were no differences in sex, BMI, and type of operation. Operation time was significantly longer in the EUG than in the non-EUG group (208.8 min vs. 121.4 min, *p* = 0.003). T3 (359.0 mg/dL vs. 188.5 mg/dL, *p* < 0.001) and FT4 (3.8 ng/dL vs. 1.7 ng/dL, *p* < 0.001) concentrations were significantly higher in the EUG than in the non-EUG group, and the percentage of patients receiving preoperative β-blockers was significantly higher in the EUG than in the non-EUG group (20% vs. 75%, *p* = 0.022).

### 3.4. Intra-Operative Changes in Vital Signs and Use of Anesthetic Agents in Patients with Extremely Uncontrolled and Non-Extremely Uncontrolled Graves’ Disease

Table 4 shows changes in vital signs and use of anesthetic agents in the EUG and non-EUG groups. There were no significant differences in the incidences of SBP increase >140 mmHg and body temperature >38 °C, and in average highest SBP, DBP, HR, and body temperature. The amount of remifentanil used was significantly higher in the EUG than in the non-EUG group (1750 µg vs. 1076 µg, *p* = 0.019).

## 4. Discussion

Patients with Graves’ disease are recommended to undergo surgery after achieving euthyroid status, as TS is more likely to occur if surgery is performed on patients with high thyroid hormone levels [7]. However, patients who experience side effects of ATDs, such as agranulocytosis and hepatotoxicity, patients unresponsive to high ATD concentrations, pregnant patients, and patients who experience symptoms accompanied by growing goiter may undergo surgery while having high thyroid hormone levels [15].

TS is defined as a severe endocrine emergency that can cause multi-organ failure. Patients diagnosed with TS are admitted to the intensive care unit for removal of triggering factors, including discontinuation of ATDs, inorganic iodide, corticosteroids, and β-adrenergic antagonists [16,17]. Although TS can occur before, during, and after surgery [18,19,20,21], its incidence is very low, at 0.2 per 100,000 people [10]. The appropriate thyroid hormone level cutoffs for TS, however, remain undetermined [5]. According to Japanese guidelines, TS is associated with FT4 levels of 3–8 ng/dL [16]. Therefore, it is necessary to determine whether vital signs change during surgery in patients whose thyroid hormone levels are and are not regulated before surgery.

Using an FT4 cutoff of 1.7 ng/dL, we found that the concentrations of T3 and FT4 and the incidence of β-blocker treatment were significantly higher in patients with FT4 ≥1.7 ng/dL (UG group) than in those with <1.7 ng/dL (CG group). However, there were no between-group differences in sex, age, vital sign change, and use of anesthetic agents during surgery. Using an FT4 cutoff of 3.4 ng/dL, we found that age was significantly lower and operation time significantly longer in patients with FT4 ≥3.4 ng/dL (EUG group) and those with <3.4 ng/dL (non-EUG group). However, we observed no significant differences in vital signs during surgery in these two groups. The total amount of remifentanil administered was significantly higher in the EUG group, because the operation time was longer. We observed that the operation time was statistically longer in the EUG group. This is thought to be due to intraoperative bleeding, but we could not obtain accurate information from the retrospective study design. This will be confirmed through follow-up research.

The absence of vital sign changes in the EUG group, with an average FT4 of 3.8 ng/dL, was not expected. The non-occurrence of TS may be due to the small number of patients, or because an FT4 level of 3.8 ng/dL was insufficient to induce TS. Because TS can more readily occur in patients with an average FT4 ≥3.4 ng/dL, it is important to reduce thyroid hormone levels as much as possible before surgery. Newly developed anesthetic agents administered by an experienced anesthesiologist may maintain stable vital signs during surgery, even for patients with high thyroid hormones.

Lugol solution is administered preoperative to shrink blood vessels and reduce the amount of bleeding during surgery [22,23]. This agent is not administered to all patients, however, because of the possibility of an allergic reaction [24]. Lugol was first used in our institution at the beginning of this study, but it was no longer available after 2015. Steroids can also be used in unregulated Graves’ and TS. According to literature reports, about 30 mg of prednisolone for 2 weeks improved symptoms of hyperthyroidism [25]. However, we have not used steroids yet.

The major limitation of this study was that we did not actually observe TS, making it impossible to compare vital sign changes in patients with and without TS. In addition, the study was retrospective in design and included a relatively small number of patients. It is therefore necessary to perform prospective, randomized trials that include larger numbers of patients. 

## 5. Conclusions

Guidelines recommend that thyroid hormone levels be normalized in patients with Graves’ disease prior to surgical treatment due to the risk of TS. Some patients, however, may undergo surgery with high thyroid hormone levels. Increases in FT4 levels to higher than the normal range and twice the normal range did not affect vital signs during surgery.

## Figures and Tables

**Table 1:** Demographic and clinical characteristics of patients with controlled and uncontrolled Graves’ disease.

	Controlled Graves’ (*n* = 12)	Uncontrolled Graves’ (*n* = 17)	*P*-value
Age, years	39.4 ± 2.66	37.4 ± 3.02	0.630
Sex (M/F)	2 / 10	2 / 15	0.706
BMI, kg/m^2^	24.2 ± 1.28	22.9 ± 0.84	0.358
Op type			0.653
Open	10	13	
Robot	2	4	
Op time, min	123.8 ± 8.17	140.3 ± 17.10	0.450
Thyroid nodule	2 (16.7%)	3 (17.6%)	0.945
T3, ng/dL	159.3 ± 10.62	247.5 ± 23.91	0.009
Preop FT4, ng/dL	1.2 ± 0.08	2.6 ± 0.19	<0.001
Preop TSH, µIU/mL	0.6 ± 0.36	0.1 ± 0.01	0.115
TSH-R-Ab, IU/L	62.6 ± 31.06	34.1 ± 11.19	0.326
Anti-thyroid drug	12 (100%)	13 (76.5%)	0.863
Methimazole	10	10	
Carbimazole	1	1	
Propylthiouracil	1	2	
Beta-Blocker	0 (0%)	8 (47.1%)	0.005
Lugol	1 (8.3%)	5 (29.4%)	0.168

BMI, body mass index; Op, operation; T3, triiodothyronine; FT4, free thyroxine; TSH, thyroid stimulating hormone; TSH-R-Ab, thyroid stimulating hormone receptor antibodies.

**Table 2:** Intra-operative changes in vital signs and use of anesthetic agents in patients with controlled and uncontrolled Graves’ disease.

	Controlled Graves’ (*n* = 12)	Uncontrolled Graves’ (*n* = 17)	*P*-value
Intra-operative changes in vital signs			
Blood pressure, mmHg SBP >140 mmHg, event	0.8 ± 0.97	0.8 ± 1.81	0.899
Highest SBP, mmHg	137.1 ± 2.87	132.9 ± 3.72	0.413
Highest DBP, mmHg	98.1 ± 5.65	88.6 ± 3.67	0.169
Peak SBP, mmHg	151	159	
Peak DBP, mmHg	115	127	
Heart rate, bpm HR >100 bpm, event	3.5 ± 1.71	2.0 ± 1.51	0.568
Highest HR, bpm	85.8 ± 11.90	96.8 ± 3.55	0.246
Peak HR, bpm	110	127	
Body temperature, °C BT >38.0 °C, event	0	0	
Highest BT, °C	36.6 ± 0.13	36.8 ± 0.96	0.330
Peak BT, °C	37.2	37.4	
Anesthetic agents			
Total propofol (mg)	61.6 ± 8.39	55.0 ± 8.38	0.595
Total remifentanil (µg)	1070.8 ± 48.64	1238.2 ± 170.51	0.428
Use of esmolel (mg)	5.8 ± 4.35	11.8 ± 6.71	0.506
Use of nicardipine (mg)	0	0.1 ± 0.06	0.411
Use of labesin (mg)	0.4 ± 0.42	0	0.241
Use of phenylephrine (ug)	2.5 ± 2.50	1.2 ± 1.18	0.603

SBP, systolic blood pressure; DBP, diastolic blood pressure; HR, heart rate; BT, body temperature.

**Table 3:** Demographic and clinical characteristics of patients with extremely uncontrolled and non-extremely uncontrolled Graves’ disease.

	None-EUG (*n* = 25)	EUG (*n* = 4)	*P*-value
Age, years	40.9 ± 1.76	21.5 ± 4.87	<0.001
Sex (M/F)	4 / 21	0 / 4	0.389
BMI, kg/m2	23.4 ± 0.76	23.5 ± 2.57	0.990
Op type			0.119
Open	12	2	
Robot	4	2	
Op time, min	121.4 ± 8.11	208.8 ± 45.20	0.003
Thyroid nodule	4 (16.0%)	1 (25.0%)	0.658
T3, mg/dL	188.5 ± 11.64	359.0 ± 61.49	<0.001
Preop FT4, ng/dL	1.7 ± 0.11	3.8 ± 0.15	<0.001
Preop TSH, µIU/mL	0.3 ± 0.18	0.1 ± 0.06	0.653
TSH-R-Ab, IU/L	40.7 ± 15.25	67.2 ± 35.21	0.480
Anti-thyroid drug	22 (88%)	3 (75%)	0.440
Methimazole	18	2	
Carbimazole	2	0	
Propylthiouracil	2	1	
Beta-Blocker	5 (20%)	3 (75%)	0.022
Lugol	5 (20%)	1 (25%)	0.819

BMI, body mass index; Op, operation; T3, triiodothyronine; FT4, free thyroxine; TSH, thyroid stimulating hormone; TSH-R-Ab, thyroid stimulating hormone receptor antibodies; EUG, extremely uncontrolled Graves’.

**Table 4:** Intra-operative changes in vital signs and use of anesthetic agents in patients with extremely uncontrolled and non-extremely uncontrolled Graves’ disease.

	Non-EUG (*n* = 25)	EUG (*n* = 4)	*P*-value
Intra-operative changes in vital signs			
Blood pressure, mmHg SBP >140 mmHg, event	0.9 ± 0.32	0	0.261
Highest SBP, mmHg	134.8 ± 2.81	133.5 ± 4.52	0.860
Highest DBP, mmHg	91.2 ± 3.43	87.8 ± 2.10	0.700
Peak SBP, mmHg	159	139	
Peak DBP, mmHg	127	92	
Heart rate, bpm HR >100 bpm, event	1.5 ± 0.61	7.3 ± 7.25	0.068
Highest HR, bpm	92.4 ± 4.43	98.8 ± 9.90	0.597
Peak HR, bpm	124	127	
Body temperature, °C BT >38.0 °C, event	0	0	
Highest BT, °C	36.7 ± 0.08	37.0 ± 0.15	0.161
Peak BT, °C	37.4	37.4	
Anesthetic agents			
Total propofol (mg)	53.6 ± 6.20	83.8 ± 14.34	0.080
Total remifentanil (µg)	1076.0 ± 87.38	1750.0 ± 434.93	0.019
Use of esmolel (mg)	6.4 ± 2.70	27.5 ± 27.5	0.091
Use of nicardipine (mg)	0.1 ± 0.04	0	0.697
Use of labesin (mg)	0.2 ± 0.20	0	0.697
Use of phenylephrine (ug)	2.0 ± 1.41	0	0.582

SBP, systolic blood pressure; DBP, diastolic blood pressure; HR, heart rate; BT, body temperature; EUG, extremely uncontrolled Graves’.
